# Effects of single-dose methylphenidate on motor performance in physically active college students with attention-deficit/hyperactivity disorder: a randomized crossover trial

**DOI:** 10.3389/fspor.2026.1869057

**Published:** 2026-08-05

**Authors:** Sharon Tsuk, Yoav Zeevi, Aviva Mimouni-Bloch

**Affiliations:** 1 The Levinsky-Wingate Academic College, Netanya, Israel; 2 Child Development Center, Loewenstein Rehabilitation Medical Center, Raanana, Israel; 3 Faculty of Medicine, Tel Aviv University, Tel Aviv, Israel

**Keywords:** agility test, balance test, motor activity, physical fitness, psychotropic drugs, sports

## Abstract

Methylphenidate (MPH) is commonly used for managing core symptoms of attention-deficit hyperactivity disorder (ADHD). However, its effects on motor performance in active individuals with ADHD have been scarcely documented. This study aimed to assess the acute effects of a single dose of MPH on agility, balance, and physiological parameters at rest and after motor tests, in physically active participants with ADHD. For this randomized crossover study, we recruited 35 physical education college students with ADHD (mean age 25.3 years; 65.7% males). Participants used their prescribed MPH medication. The following evaluations were performed both before and after MPH treatment: motor performance tests (a zigzag agility test, both with and without leading a ball; and a balance test), physiological measurements (heart rate, blood pressure, core temperature) recorded at rest and after the motor tests (at least one week apart); mental evaluations using a rated perceived exertion (RPE) scale. We found no significant differences in motor performance, physiological measures, or RPE with vs. without MPH. These results suggest that while MPH may enhance certain motor functions as shown in other studies, such effects are not universally beneficial across all motor performance measures in young physically active adults diagnosed with ADHD.

**Clinical Trial Registration:** Clinicaltrials.gov, identifier NCT04283604.

## Introduction

Methylphenidate (MPH) is widely recognized as the most common pharmacological treatment for attention-deficit hyperactivity disorder (ADHD), primarily targeting core symptoms such as hyperactivity, impulsivity, and inattention (1, 2). Its therapeutic effects are largely attributed to the inhibition of dopamine reuptake, which enhances dopaminergic signaling in the brain (3). Despite these cognitive benefits, the effects of MPH on other functional domains, particularly motor performance during physical activity, remain less understood. Children and adolescents with ADHD have been shown to exhibit a significantly higher risk of motor difficulties, including impairments in balance, locomotion, and object control skills (4). Consequently, understanding the effects of MPH on motor abilities in adults with ADHD is of particular importance.

MPH exerts its effects primarily by blocking dopamine and norepinephrine transporters, which reduces catecholamine reuptake and increases their synaptic availability in neural circuits involved in cognitive and motor control, especially within the prefrontal cortex, basal ganglia (including the striatum) and cerebellum (5). Enhanced dopaminergic transmission within the basal ganglia facilitates motor initiation, sequencing of actions, and rapid switching between movement patterns, which are essential for agility tasks that require fast directional changes (6–8). Within the prefrontal cortex, increased catecholamine signalling improves attentional control, working memory, and inhibition of competing motor responses, allowing more accurate motor planning and cognitive–motor coordination during high-demand tasks Maki& McIlroy (9),. In addition, elevated catecholamine activity within cerebellar circuits supports sensorimotor integration, postural adjustments, and fine-tuning of limb trajectories, contributing to more stable and adaptive movement under conditions requiring dynamic balance (10).

MPH may exert distinct motor benefits in individuals with ADHD due to the characteristic disruptions in neural systems underlying attention, motor coordination, and executive–motor integration. ADHD is associated with altered dopamine transmission within fronto-striatal and cerebello-thalamo-cortical networks. This contributes not only to inattention and impulsivity but also to deficits in movement sequencing, postural control, and balance Castellanos & Proal (11),. A recent review (12) indicated that motor difficulties in ADHD reflect disruptions in action systems. Furthermore, the authors found that motor difficulties and cognitive challenges in ADHD may co-emerge from the same underlying disruptions in sensorimotor processes and environmental exploration, rather than existing in a unidirectional or secondary relationship (12). Because MPH enhances catecholamine signalling specifically within these dysregulated pathways, individuals with ADHD may demonstrate larger improvements in agility and dynamic balance compared with those whose motor systems are already optimized. Therefore, examining the motor effects of MPH in ADHD provides insight into how pharmacological normalization of executive-motor circuitry may translate into enhanced physical performance under cognitively demanding conditions.

Emerging evidence indicates that MPH has positive impacts on exercise performance. For example, studies have shown that MPH can improve gait function in older adults (13–15). In a double-blind crossover study, healthy young participants ingested 20 mg of immediate release MPH or placebo 90 min before performing a muscle-fatiguing handgrip task during functional magnetic resonance imaging (16). Under the MPH challenge, participants increased grip force throughout the task. Additionally, brain connectivity was altered throughout the task between the insular and the hand motor cortex, as well as between the insular and the orbital frontal cortex (16). MPH has also exhibited ergogenic effects in well-trained male cyclists under heated conditions, although these benefits were not observed in temperate environments (17). In elite cyclists, MPH administration resulted in higher power output, oxygen consumption, heart rate, ventilatory volume, and blood lactate levels, despite no changes recorded in electromyographic activity (18). MPH demonstrated an effect on measures of physical performance in terms of power output and exercise performance in timed trials in athletes (19). A recent, well conducted systemic review concluded that “there is support for the presumption that stimulant medications impact athletic performance” (19).

Agility, defined as the rapid and accurate ability to change direction, is an important component of motor skills required for sport and physical exercise (20). Successful performance in agility tasks requires the integration of sensorimotor input, motor coordination, the ability to accelerate and decelerate, and executive control processes, including attention, motor planning, and precise timing (21). Meta-analytic evidence indicates that deficits in these domains are prevalent and robust across development in individuals with ADHD (4). Such difficulties have been linked to abnormalities in dopaminergic signaling within cortico-striatal pathways involved in both cognitive and motor control processes (22). MPH has been shown to improve motor timing, coordination, and postural stability in both children and adults with ADHD (23, 24). These findings suggest that stimulant medication may enhance neural processes underlying decision-movement coupling and sensorimotor integration. Because agility relies on these same mechanisms, examining agility under methylphenidate treatment provides a theoretically grounded approach for understanding how pharmacological modulation of dopaminergic function may influence motor coordination in individuals with ADHD.

In ADHD, altered dopaminergic signaling within these pathways contributes to delayed reaction times, reduced efficiency of motor switching, and greater variability in change-of-direction performance (25, 26). By enhancing dopamine availability in the basal ganglia and prefrontal cortex, MPH may normalize these neural processes, potentially reduce motor hesitancy, and improve decision-movement coupling during high-speed directional transitions. Furthermore, the attentional control required to anticipate and adapt to rapidly changing movement demands is impaired in ADHD but has been shown to improve following MPH-induced catecholamine modulation in the prefrontal cortex (8). Together, these mechanisms provide a strong theoretical rationale for examining whether MPH preferentially enhances agility performance in individuals with ADHD, for whom deficits in executive-motor integration may render this motor skill particularly sensitive to pharmacological modulation.

Most previous studies examining MPH and motor performance focused on children, older adults, athletes, and/or assessed controlled fixed-dose protocols, while less is known about the acute motor and physiological effects of routinely prescribed MPH in physically active young adults with ADHD. The present study addresses this gap by examining agility, ball-related agility, balance, and physiological responses in a real-world crossover setting in which each participant served as their own control.

### Objectives and hypothesis

Given the widespread use of MPH among individuals with ADHD, it is important to further explore its impact on exercise performance across various physical activities. This study aimed to investigate the effects of MPH on performance in agility and balance tasks, which are essential components of many sports and physical activities. Specifically, we evaluated the effects of a single dose of MPH on agility, agility while dribbling a ball, and balance tasks in young, physically active college students diagnosed with ADHD, by comparing their performance with and without MPH. We hypothesized that a single dose of MPH, as tested in this study, would lead to significant performance benefits across the assessed motor tasks, and that these effects would be most evident in tasks involving greater attentional control and cognitive-motor integration, particularly agility while dribbling a ball.

## Methods

### Participants

A total of 53 healthy physical education college students with an ADHD diagnosis were recruited, of which 35 completed the study. The data of the 35 participants were analyzed and are presented here. The mean ± standard deviation (SD) age of the participants who completed the study was 25.3 ± 3.6 years, and 23 (65.7%) of them were males. Demographic and clinical baseline characteristics, as summarized in Table 1, were collected at the laboratory. All participants were from the same physical education college program, and they were all recruited between April 2023 and March 2024. Eighteen participants did not complete the study, including one who withdrew upon discovering she was pregnant, two who sustained injuries unrelated to the study, and 15 who declined to attend the second meeting, citing the need to arrive on campus earlier than usual. Potential attrition bias was assessed by comparing available demographic, anthropometric, and clinical variables between participants who completed the study (*n* = 35) and those who withdrew (*n* = 18). Examination of sex, age, body measures, medication status, and baseline Adult ADHD Self-Report Scale (ASRS) scores revealed substantial overlap between groups, with no evidence of systematic attrition related to baseline characteristics.

**Table 1:** Demographic and baseline characteristics of study participants (*N* = 35). Values are presented as numbers (percentages) or mean ± SD.

Demographic/baseline characteristic (*N* = 35)	Statistical value
Sex (male), *n* (%)	23 (65.7%)
Sex (female), *n* (%)	12 (34.3%)
Weight (kilograms), mean (SD)	71.3 ± 11.3
Height (meters), mean (SD)	1.7 ± 0.1
Age (years), mean (SD)	25.3 ± 3.6
BMI, mean (SD)	23.7 ± 2.6
Stimulant frequency, *n* (%)	
Every day	10 (28.6%)
2–5 times a week	8 (22.9%)
Not regularly, when needed	16 (45.7%)
Type of MPH, *n* (%)	
Short-acting MPH	12 (34.3%)
Intermediate-acting MPH	15 (42.9%)
Long-acting MPH	8 (22.9%)

BMI, body mass index; MPH, methylphenidate; SD, standard deviation.

For stimulant frequency there was one missing value, and therefore, the statistical analysis was carried out from a total of 34, not 35 values.

In order to be recruited, candidates had to meet the following main eligibility criteria: students of physical education formally diagnosed with ADHD and taking one of the following prescription stimulant MPH derivative medications: short-acting Ritalin, Ritalin sustained release, long-acting Ritalin, Concerta.

The participants' ADHD diagnosis was accepted only if it had been made by a neurologist, psychiatrist, or other ADHD physician specialist. All participants had been treated with MPH at some point during the five years prior to the start of the study. Participants reported their regular use of MPH prior to the start of the study. They were asked whether they typically took MPH daily during the week or whether there were times when they preferred not to use it. Most participants (*n* = 24) reported using MPH only occasionally, when they felt it was needed and based on their own decision. Seven participants reported avoiding MPH when they anticipated engaging in physical activity, one participant reported not taking MPH when ill, and three participants reported taking MPH consistently.

Any participant with self-reported chronic illness other than ADHD (e.g., asthma, gastrointestinal disorders, depression, anxiety, etc.) was excluded.

### Procedure

For this study, a crossover randomized design was used to assess the effects of MPH on agility and balance motor tests. This design ensured that each participant served as their own control, thereby minimizing inter-individual variability and enhancing the internal validity of the study.

Participants were recruited through emails and class announcements at a physical education college. During the first session with the recruiting team, candidates received a comprehensive overview of the study's objectives and procedures, ensuring they were fully informed before commencing the trial. Next, they were instructed to sign an informed consent form approved by the Institutional Review Board (Loewenstein Rehabilitation Medical Center, 0007–19-LOE), and to fill out a general background questionnaire (see below) and an ADHD self-report scale (ASRS) questionnaire.

The order of sessions with or without MPH treatment was randomly decided, resulting in 23 participants starting with the session without medication, and 12 participants starting with the session with medication. To prevent bias, the session order was randomized via a lottery system by a research team member who was not involved in conducting the motor tests. Each participant was then assessed at two additional, second and third sessions. The assessments in these two sessions consisted of motor tests (see below), which were conducted at least one week apart at a fitness room in the physical education college campus. In one of these two sessions, participants were asked to arrive at the session before they took their MPH medication, while in the other session, they were asked to arrive after consuming their usual MPH medication (1 h before the start of the session). For the session conducted after MPH intake, the participants took their treatments themselves as prescribed by their physicians for their routine usage. Importantly, using prescribed medication ensures individual appropriateness in terms of both type and dosage. We considered this approach to be more ecologically valid than a traditional placebo-controlled design.

### Study outcomes

The primary outcome of the study was the change in motor performance after MPH intake compared to without MPH intake, as measured via the balance test and the agility tests. The secondary outcomes of the study were the changes in physiological variables after MPH intake compared to without MPH intake. The list of physiological variables, as well as a description of the motor tests used are provided below.

### Background questionnaire

The background questionnaire consisted of general information relevant to this study, which included sex (referring to the biological attributes of participants), anthropometrical characteristics, chronic illnesses, regular medication consumption, ADHD diagnosis, MPH consumption, and physical activity participation.

### Assessment of ADHD symptoms

ADHD symptoms were assessed using the Hebrew version of the ASRS questionnaire, which has demonstrated good psychometric properties in Israeli adults. The ASRS includes 18 items rated on a 5-point Likert scale, comprising a 6-item screening subscale (Part A) and a 12-item supplementary subscale (Part B). In accordance with prior validation studies, summed scores were calculated for Part A, Part B, and the total ASRS score (all 18 items). Given the absence of an established diagnostic cut-off for summed scores, ASRS results were analyzed as continuous measures reflecting symptom burden rather than categorical ADHD diagnosis (27).

### Motor tests

Before each testing session, participants completed a brief standardized warm-up, consisting of stretching. Uniform rest intervals were maintained between tests, and for each participant, the same assessor conducted both testing sessions. Participants performed the tests in the same sports hall and wore the same shoes during both sessions to reduce measurement variability.

### Balance test

The participants were requested to perform a one leg blind balance test. The maximum number of seconds a participant was able to stand without toppling over or placing a foot on the floor was recorded. Participants stood on both legs with their eyes open, and then lifted one leg with their eyes closed, facing forward on a marked site. The time recording stopped when participants began to teeter, put their foot on the floor, or opened their eyes (28). Two attempts (one for each leg) were made, and the higher value of these attempts was recorded.

#### Agility tests

Two agility tests (zigzag with and without a ball) were performed twice, the best time of each test was recorded, and the ratio of the zigzag test scores with and without the ball was calculated. The two agility tests used in this study had been evaluated and validated elsewhere (29).

1. **Zigzag test** (running agility test): Participants were required to run a zigzag course in the shortest possible time. The zigzag course consisted of a rectangle with dimensions of 3 × 5 meters, which was set out at a 100° angle by four cones placed in the corners, with one more cone placed in the center. Overall, there were six segments numbered from 1 to 6 according to their order in the test. This test measures rapid acceleration, deceleration, and balance control (29–31).
2. **Zigzag agility drill with a ball:** The ability to control the ball while changing direction was assessed. Subjects were instructed to run with the ball as fast as possible along the same zigzag path used in the previous test. This test was similar to the zigzag test, except for the fact that the participants had to perform it while leading a ball with their legs (32). This test measures agility, rapid acceleration, deceleration, as well as balance and ball control.

### Measurements of physiological variables

#### Blood pressure (BP)

BP was measured at the beginning of each session after sitting for at least 5 min. Peak BP was measured at the end of the last agility test at each session. BP was measured using a mercury sphygmomanometer.

#### Body temperature

Core body temperature was measured sublingually at the beginning of each session after sitting for at least 5 min. Peak blood temperature was measured at the end of the last agility test at each session. Core body temperature was measured using an electronic thermometer.

#### Heart rate

Resting heart rate was recorded at the beginning of each session after sitting for at least 5 min and at the end of the last agility test. Heart rate was measured by polar sensor chest straps and a watch (Polar heart rate monitor S710).

#### Borg rate of perceived exertion (RPE)

The Borg RPE was measured using the RPE scale (33) at the end of the last agility test at each session. The scale measures subjective sense of effort using a range between 6 and 20.

### Statistical analysis

The statistical analyses were performed by a statistician who was not involved in the motor testing and who analyzed coded data. As the study used a randomized crossover design, each outcome was analyzed as a within-participant paired comparison between the MPH and no-MPH sessions. Descriptive statistics are presented as median (Q1, Q3). The primary inferential analysis used two-sided Wilcoxon signed-rank tests.

To control for multiplicity while preserving the distinction between the prespecified outcome groups, the Benjamini-Hochberg false-discovery-rate correction was applied separately within the motor-performance group (agility, agility with ball, and balance) and within the physiological/RPE group (blood pressure, heart rate, core temperature, and RPE outcomes). Raw Wilcoxon *p*-values are reported for transparency; however, statistical significance was interpreted using the BH-adjusted q-value within the corresponding outcome group. This group-structured approach is consistent with the TreeBH-style multiplicity control.

Paired mean differences, 95% confidence intervals, and Cohen's dz are reported descriptively to provide numeric effect-size information. Parametric paired t analyses were conducted as sensitivity analyses and did not alter the substantive conclusions.

Analyses used complete paired data only; no imputation was performed. The paired complete-case set included 35 participants for all outcomes. No subgroup or moderator analyses according to sex, age, medication formulation, dose, or medication-use frequency were performed because the study was not powered for reliable subgroup inference and the sample size within each subgroup would have been small and unequal.

All statistical analyses were carried out using R-4.4.1 (R Foundation for Statistical Computing, Vienna, Austria).

The sample size was calculated to compare the difference in agility with the ball between two time points: with and without MPH, using a paired t-test. Based on prior data from the literature, the mean agility time was assumed to be 8.8 s without MPH and 8.2 s with MPH, corresponding to a mean difference of 0.6 s. A SD of the mean difference of 0.7 s was assumed. The calculation was performed under the assumption of a normal distribution, using a two-sided paired t-test, with a significance level (*α*) of 0.05 and 90% power (1–*β* = 0.90). Under these assumptions, the required sample size was estimated to be 17 participants. Allowing an anticipated 15% dropout rate, we planned to recruit at least 20 participants.

### Trial registration

The study is registered at the local Ministry of Health (registration number MOH_2019-12-03_008534), and at the ClinicalTrials.gov registry (ID NCT04283604; https://www.clinicaltrials.gov/study/NCT04283604; registered on 22 February 2020). The full study protocol and statistical analysis plan are available upon request from the corresponding author. The study was performed in accordance with the Declaration of Helsinki. Informed consent was obtained for all participants. The results of this study are presented clearly, honestly, and without fabrication, falsification, or inappropriate data manipulation.

## Results

As aforementioned, the ADHD symptom severity was assessed using the ASRS. Participants demonstrated moderate to high ADHD symptom severity levels, with mean (SD) scores of 18.9 ± 3.9 for Part A, 37.1 ± 6.8 for Part B, and a total score of 56.0 ± 9.6.

Motor test results (agility, agility with a ball and balance) did not show a statistically significant improvement in performance under MPH condition compared to the no-MPH condition; see Table 2.

**Table 2:** Motor test results with and without a single dose of MPH (*N* = 35).

Outcome	Without MPH median (Q1, Q3)	With MPH median (Q1, Q3)	Paired mean difference (95% CI)	Cohen's dz	Raw p	BH q
Agility (sec)	7.4 (6.9, 7.8)	7.2 (6.8, 7.7)	−0.13 (−0.24, −0.01)	−0.37	0.062	0.186
Agility with ball (sec)	13.6 (10.6, 16.9)	13.2 (11.2, 18.1)	0.21 (−0.29, 0.70)	0.14	0.204	0.204
Balance (sec)	24.1 (12.0, 62.0)	25.1 (13.3, 67.8)	4.46 (−6.23, 15.15)	0.14	0.130	0.195

Values are presented as median (Q1, Q3). Paired mean differences are calculated as “with MPH” minus “without MPH”.

CI, confidence interval; MPH, methylphenidate.

BH q = represents the Benjamini-Hochberg adjusted q-value within the prespecified motor-performance outcome group. Paired mean difference = “with MPH” minus “without MPH”. Paired mean differences, 95% CIs, and Cohen's dz are based on paired differences and are reported descriptively. Raw *p*-values and BH q-values are based on Wilcoxon signed-rank tests. Parametric paired- t sensitivity analyses did not alter the substantive conclusions. For agility outcomes, negative paired mean differences indicate faster performance with MPH.

Physiological parameters were assessed with and without MPH at rest and after the motor tests and results are presented in Table 3. No statistical differences were observed in these assessments between the two conditions (with and without MPH). Similarly, perceived effort after agility tests was also comparable across conditions.

**Table 3:** Physiological and perceived-exertion outcomes with and without a single dose of MPH (*N* = 35).

Outcome	Without MPH median (Q1, Q3)	With MPH median (Q1, Q3)	Paired mean difference (95% CI)	Cohen's dz	Raw p	BH q
Resting SBP (mmHg)	126.0 (120.0, 134.0)	130.0 (120.5, 143.5)	5.26 (0.66, 9.86)	0.39	0.033	0.465
SBP after agility (mmHg)	143.0 (133.0, 158.5)	146.0 (137.0, 163.5)	3.71 (−0.92, 8.35)	0.28	0.143	0.666
SBP after agility with ball (mmHg)	149.0 (135.5, 167.5)	150.0 (139.5, 158.0)	−1.60 (−7.93, 4.73)	−0.09	0.617	0.846
Resting DBP (mmHg)	76.0 (71.5, 80.5)	79.0 (72.0, 86.0)	1.29 (−2.14, 4.71)	0.13	0.471	0.846
DBP after agility (mmHg)	77.0 (73.5, 86.5)	80.0 (75.0, 86.5)	−0.14 (−3.70, 3.41)	−0.01	0.905	0.905
DBP after agility with ball (mmHg)	83.0 (77.0, 87.0)	81.0 (75.0, 89.5)	−0.57 (−4.58, 3.44)	−0.05	0.714	0.846
Resting heart rate (bpm)	68.0 (64.5, 78.0)	71.0 (66.0, 79.0)	3.94 (−1.35, 9.23)	0.26	0.080	0.561
Heart rate after agility (bpm)	132.0 (124.0, 146.5)	136.0 (125.0, 145.0)	1.34 (−3.12, 5.81)	0.10	0.321	0.846
Heart rate after agility with ball (bpm)	137.0 (125.5, 147.0)	135.0 (129.5, 147.0)	1.63 (−2.69, 5.94)	0.13	0.481	0.846
Resting core temperature (°C)	36.6 (36.5, 36.8)	36.6 (36.5, 36.8)	0.04 (−0.05, 0.13)	0.16	0.229	0.803
Core temperature after agility (°C)	36.6 (36.5, 36.8)	36.7 (36.6, 36.8)	−0.00 (−0.10, 0.10)	−0.01	0.637	0.846
Core temperature after agility with ball (°C)	36.6 (36.5, 36.7)	36.6 (36.5, 36.7)	−0.02 (−0.11, 0.07)	−0.08	0.785	0.846
RPE after agility	11.0 (8.5, 12.0)	11.0 (8.0, 11.0)	−0.34 (−1.02, 0.34)	−0.17	0.485	0.846
RPE after agility with ball	11.0 (10.0, 13.0)	11.0 (10.0, 13.0)	−0.20 (−1.03, 0.63)	−0.08	0.775	0.846

Values are presented as median (Q1, Q3). Paired mean differences are calculated as “with MPH” minus “without MPH”.

bpm, beats per minute; CI, confidence interval; DBP, diastolic blood pressure; MPH, methylphenidate; RPE, rate of perceived exertion; SBP, systolic blood pressure.

BH *q* = represents the Benjamini-Hochberg adjusted q-value within the prespecified motor-performance outcome group. Paired mean difference = “with MPH” minus “without MPH”. Paired mean differences, 95% CIs, and Cohen's dz are based on paired differences and are reported descriptively. Raw *p*-values and BH q-values are based on Wilcoxon signed-rank tests. Parametric paired- t sensitivity analyses did not alter the substantive conclusions. For agility outcomes, negative paired mean differences indicate faster performance with MPH.

## Discussion

The present study examined the acute effects of a single dose of MPH on agility, agility while leading a ball, and balance in physically active young adults with ADHD. Contrary to our hypothesis, MPH did not lead to significant performance benefits across any of these motor tasks. Although the selected tests were designed to be sufficiently sensitive to detect potential medication-related effects, including tasks with differing cognitive–attentional demands, the findings suggest that a single dose of MPH does not substantially enhance motor performance in the tested population. Taken together, these findings suggest that the acute effects of MPH on motor performance are likely task- and context-dependent. A possible interpretation is that MPH-related enhancement of attention and executive control may be more relevant for tasks in which performance is primarily limited by cognitive regulation, timing, or motor variability, whereas the present field-based tasks also depend strongly on physical execution, task familiarity, and automatized motor control. Based on this hypothesis, the absence of significant performance gains does not necessarily indicate that MPH has no effects on motor performance, but rather that such effects may be small, task-specific, or not detectable in physically active young adults performing relatively familiar motor tasks.

Our hypothesis was grounded in established models of attentional control and executive–motor integration, which posit that motor tasks with higher cognitive demands rely more heavily on prefrontal–basal ganglia circuitry and dopaminergic modulation. Executive control is engaged during intentional, goal-directed processing via top-down regulation by lateral prefrontal regions, in interaction with dopaminergic gating mechanisms of the basal ganglia (34). Friedman and Robbins (35) describe cognitive control and executive function as processes that support goal-directed behaviour rather than habitual or automatic responses, implicating the prefrontal cortex and its networks in the controlled processing demanded by cognitively challenging tasks (35).

In the current study, tasks requiring concurrent motor execution and cognitive monitoring, such as agility while leading a ball, were expected to benefit most from MPH, as these tasks place greater demands on prefrontal cognitive control processes that are known to be enhanced by catecholaminergic modulation following MPH administration (36). However, the absence of performance improvements in these tasks suggests that, among physically active young adults with ADHD, performance in these relatively familiar motor tasks may rely less on acute changes in executive control processes than initially expected. In this context, additional dopaminergic modulation via MPH may confer limited benefit, consistent with the inverted-U model of dopamine function and the law of diminishing returns (37). This outcome supports previous reports demonstrating heterogeneous and task-specific effects of MPH on motor coordination and complex motor skills, which appear to vary across populations, baseline motor proficiency, and task characteristics (14, 16, 38), and is particularly evident in adult and physically active subjects (39, 40).

Balance impairments have been frequently reported in individuals with ADHD. Children with ADHD demonstrate poorer stability and abnormal sensory integration underlying balance function compared to typically developing peers, including deficits in somatosensory, visual, and vestibular integration (41). Similar findings extend into adulthood, with adults with ADHD exhibiting significantly greater postural sway than healthy controls (42). Contrary to this, no significant differences in balance outcomes were observed between MPH and no-MPH conditions in the present study. One explanation for this finding is that a single dose of MPH may not be effective in modifying balance performance in physically active young adults, even among individuals who may exhibit relative balance deficits associated with ADHD. Importantly, beneficial effects of MPH on motor performance have been reported primarily in older adults and non–physically active populations, including improvements in gait function and postural stability (43), as well as increased hand grip strength in young adults (16). Our sample, however, consisted of physically active young adults with ADHD, who likely exhibit high baseline motor proficiency and efficient executive–motor integration. We postulate that such characteristics reduce responsiveness to pharmacological enhancement, particularly when motor tasks are already well learned and largely automatized.

Previous studies reporting beneficial effects of MPH on motor performance have predominantly employed tasks with relatively low biomechanical demands and a substantial reliance on executive and attentional control, such as gait regulation, motor coordination, or variability-based outcomes. In such paradigms, the motor component itself is mechanically simple, while performance is constrained primarily by cognitive monitoring, timing, and executive regulation. For example, Bart et al. (39) assessed motor performance using standardized coordination tasks that emphasize planning, sequencing, and controlled execution rather than speed or force production (39). Gait performance has been shown to deteriorate under dual-task conditions, particularly when the motor task itself is more challenging, reflecting increased cognitive–motor interference as task demands increase (44). Möhring et al. (45) examined gait variability during steady-state walking under cognitive load, a task characterized by minimal mechanical challenge but increased executive control demands (45). In these contexts, improvements following MPH administration are believed to reflect enhanced top-down cognitive control over motor output.

In contrast, the agility and balance tasks used in the present study involved rapid, time-constrained motor execution, potentially placing greater emphasis on automatized motor control and physical capacity. While this interpretation remains speculative, it is possible that such task characteristics reduce sensitivity to acute pharmacological modulation of executive processes. Additionally, differences in medication protocols, including chronic vs. acute MPH administration, may also contribute to inconsistent findings across studies. Taken together, these distinctions provide a coherent framework for understanding the heterogeneity of findings regarding the effects of MPH on motor performance.

The physically active nature of our sample may be a critical moderating factor. According to the law of diminishing returns, individuals with high baseline performance levels may experience limited additional benefits from interventions aimed at enhancing cognitive or motor function. More specifically, well-trained individuals often show smaller adaptation gains from standardized interventions compared with less-trained individuals, requiring more demanding stimuli to elicit further improvements (46). This observation has important practical implications, as physically active individuals with ADHD may intentionally refrain from taking MPH before training or competition out of concern for potential effects on physical performance. Supporting this notion, recent work from our group demonstrated that 94.1% of physical education college students with ADHD reported intentionally skipping prescribed medication at certain times (47). Similarly, in the current study, seven participants reported avoiding their MPH medication before engaging in physical activity.

Notably, our secondary physiological outcomes (heart rate, core temperature, and perceived exertion) did not differ between conditions, aligning with evidence that acute MPH effects on physiological strain during exercise can be small or context-dependent, such as exercising in heat conditions vs. normal temperature. In a prolonged exercise in a hot environment, acute MPH administration improved time-trial performance and increased core temperature without any change in ratings of perceived exertion or thermal stress compared with placebo. This suggests potential dissociation between physiological strain and subjective effort under stimulant administration Roelands et al. (17).

### Limitations

Several limitations of this study should be noted. First, the sample consisted of physically active college students with ADHD enrolled in a physical education program, which may limit the generalizability of the findings to the broader ADHD population. Although these participants were not competitive athletes or highly trained sports performers, their familiarity with basic motor tasks may have led to performance gains that were lower than the detection threshold of the selected agility and balance tests, thus creating a ceiling effect. Second, participants self-administered their medication according to their usual prescriptions, which may have introduced variability in dosing and timing. Administration under controlled clinical conditions could provide more precise estimates of MPH effects on motor performance. Although all participants were assessed approximately 1 h after medication intake, the prescribed MPH formulations differed across participants. Because short-, intermediate-, and long-acting formulations differ in onset and peak plasma concentrations, participants may not have been tested at comparable pharmacokinetic phases. This heterogeneity may have attenuated medication-related effects, which limits mechanistic interpretation of the findings. Third, this study used a pragmatic, ecologically valid crossover design in which participants self-administered their routinely prescribed MPH medication, and was not placebo-controlled or blinded. Therefore, expectancy and nocebo/placebo effects cannot be excluded, particularly for subjective outcomes such as RPE and for voluntary maximal-effort performance. For this reason, findings should be interpreted as reflecting performance under real-world medication-use conditions rather than as evidence of isolated pharmacological effects of MPH. Fourth, comorbidity was self-reported and there were no formal assessments for mental conditions, which may introduce biases and inaccuracy of data.

## Conclusions and future directions

Our findings do not support significant performance benefits of a single dose of MPH on agility and balance tasks in young, physically active adults with ADHD. Under the specific conditions examined, MPH did not confer measurable improvements in motor performance. While MPH is well established as an effective pharmacological treatment for core ADHD symptoms, the present results do not support an acute ergogenic effect of a single dose of MPH on agility or balance in this specific context.

Future studies are warranted to further elucidate the effects of MPH on motor performance across a broader range of conditions. Research involving larger and more diverse samples, including individuals with varying levels of physical activity, different age groups, sex, and distinct ADHD profiles may help clarify potential subgroup specific effects. In addition, studies examining chronic MPH administration, controlled formulations and dosing, and a wider range of motor tasks with differing cognitive and executive demands may provide deeper insight into the circumstances under which MPH could influence motor performance Supplementary Figure S1.

## Data Availability

The raw data supporting the conclusions of this article will be made available by the authors, without undue reservation.

## References

[1] Ma F. Diagnostic and statistical manual of mental disorders-5 (DSM-5). In: Gu D Dupre ME, editors. Encyclopedia of Gerontology and Population Aging. Cham: Springer International Publishing (2020). p. 1–12.

[2] Huss M Duhan P Gandhi P Chen CW Spannhuth C Kumar V. Methylphenidate dose optimization for ADHD treatment: review of safety, efficacy, and clinical necessity. Neuropsychiatr Dis Treat. (2017) 13:1741–51. 10.2147/NDT.S130444 28740389 PMC5505611

[3] Zimmer L. Contribution of clinical neuroimaging to the understanding of the pharmacology of methylphenidate. Trends Pharmacol Sci. (2017) 38:608–20. 10.1016/j.tips.2017.04.001 28450072

[4] Blanco-Martínez N González-Devesa D Ayán-Pérez C Diz-Gómez JC. Differences in motor competence between children and adolescents with and without ADHD: findings from a systematic review and meta-analysis. J Autism Dev Disord. (2025):1–15. 10.1007/s10803-025-07033-1 40913697

[5] Berridge CW Devilbiss DM Andrzejewski ME Arnsten AFT Kelley AE Schmeichel B. Methylphenidate preferentially increases catecholamine neurotransmission within the prefrontal Cortex at low doses that enhance cognitive function. Biol Psychiatry. (2006) 60:1111–20. 10.1016/j.biopsych.2006.04.022 16806100

[6] Phillips CD Hodge AT Myers CC Leventhal DK Burgess CR. Striatal dopamine contributions to skilled motor learning. J Neurosci. (2024) 44:e0240242024. 10.1523/JNEUROSCI.0240-24.2024 38806248 PMC11211718

[7] Klaus A Alves Da Silva J Costa RM. What, if, and when to move: basal ganglia circuits and self-paced action initiation. Annu Rev Neurosci. (2019) 42:459–83. 10.1146/annurev-neuro-072116-031033 31018098

[8] Arnsten AFT. Catecholamine influences on dorsolateral prefrontal cortical networks. Biol Psychiatry. (2011) 69:e89–99. 10.1016/j.biopsych.2011.01.027 21489408 PMC3145207

[9] Maki BE McIlroy WE. Cognitive demands and cortical control of human balance-recovery reactions. J Neural Transm. (2007) 114:1279–96. 10.1007/s00702-007-0764-y 17557125

[10] Yoshida J Oñate M Khatami L Vera J Nadim F Khodakhah K. Cerebellar contributions to the basal ganglia influence motor coordination, reward processing, and movement vigor. J Neurosci. (2022) 42:8406–15. 10.1523/JNEUROSCI.1535-22.2022 36351826 PMC9665921

[11] Castellanos FX Proal E. Large-scale brain systems in ADHD: beyond the prefrontal-striatal model. Trends Cogn Sci. (2012) 16:17–26. 10.1016/j.tics.2011.11.007 22169776 PMC3272832

[12] Shimko GA James KH. The relationship between motor development and ADHD: a critical review and future directions. Behav Sci. (2025) 15:576. 10.3390/BS15050576 40426354 PMC12108854

[13] Shorer Z Bachner Y Guy T Melzer I. Effect of single dose methylphenidate on walking and postural stability under single- and dual-task conditions in older adults–A double-blind randomized control trial. J Gerontol A Biol Sci Med Sci. (2013) 68:1271–80. 10.1093/gerona/glt035 23580740

[14] Ben-Itzhak R Giladi N Gruendlinger L Hausdorff JM. Can methylphenidate reduce fall risk in community-living older adults? A double-blind, single-dose cross-over study. J Am Geriatr Soc. (2008) 56:695–700. 10.1111/j.1532-5415.2007.01623.x 18266665 PMC2441772

[15] Cordeiro LMS Rabelo PCR Moraes MM Teixeira-Coelho F Coimbra CC Wanner SP. Physical exercise-induced fatigue: the role of serotonergic and dopaminergic systems. Braz J Med Biol Res. (2017) 50:e6432. 10.1590/1414-431X20176432 29069229 PMC5649871

[16] King M Rauch LHG Brooks SJ Stein DJ Lutz K. Methylphenidate enhances grip force and alters brain connectivity. Med Sci Sports Exerc. (2017) 49:1443–51. 10.1249/MSS.0000000000001252 28277403

[17] Roelands B Hasegawa H Watson P Piacentini MF Buyse L De Schutter G. The effects of acute dopamine reuptake inhibition on performance. Med Sci Sports Exerc. (2008) 40:879–85. 10.1249/MSS.0b013e3181659c4d 18408610

[18] Swart J Lamberts RP Lambert MI St Clair Gibson A Lambert EV Skowno J. Exercising with reserve: evidence that the central nervous system regulates prolonged exercise performance. Br J Sports Med. (2009) 43:782–8. 10.1136/bjsm.2008.055889 19052141

[19] Berezanskaya J Cade W Best TM Paultre K Kienstra C. ADHD Prescription medications and their effect on athletic performance: a systematic review and meta-analysis. Sports Med Open. (2022) 8:article no. 5. 10.1186/S40798-021-00374-Y 35022919 PMC8755863

[20] Sheppard J Young W. Agility literature review: classifications, training and testing. J Sports Sci. (2006) 24:919–32. 10.1080/02640410500457109 16882626

[21] Wilson J Sup M Wilson M Maillet MA Mekary S. Developing speed qualities in youth athletes. In: Porter S Wilson J, editors. A Comprehensive Guide to Sports Physiology and Injury Management: An Interdisciplinary Approach. London: Elsevier (2021). p. 411–9.

[22] del Campo N Fryer TD Hong YT Smith R Brichard L Acosta-Cabronero J. A positron emission tomography study of nigro-striatal dopaminergic mechanisms underlying attention: implications for ADHD and its treatment. Brain. (2013) 136:3252–70. 10.1093/brain/awt263 24163364 PMC4125626

[23] Brossard-Racine M Shevell M Snider L Bélanger SA Majnemer A. Motor skills of children newly diagnosed with attention deficit hyperactivity disorder prior to and following treatment with stimulant medication. Res Dev Disabil. (2012) 33:2080–7. 10.1016/j.ridd.2012.06.003 22796639

[24] Jacobi-Polishook T Shorer Z Melzer I. The effect of methylphenidate on postural stability under single and dual task conditions in children with attention deficit hyperactivity disorder—a double blind randomized control trial. J Neurol Sci. (2009) 280:15–21. 10.1016/J.JNS.2009.01.007 19217632

[25] Kofler MJ Rapport MD Sarver DE Raiker JS Orban SA Friedman LM. Reaction time variability in ADHD: a meta-analytic review of 319 studies. Clin Psychol Rev. (2013) 33:795–811. 10.1016/j.cpr.2013.06.001 23872284

[26] Klotz JM Johnson MD Wu SW Isaacs KM Gilbert DL. Relationship between reaction time variability and motor skill development in ADHD. Child Neuropsychol. (2012) 18:576–85. 10.1080/09297049.2011.625356 22111593 PMC3461120

[27] Zohar AH Konfortes H. Diagnosing ADHD in Israeli adults: the psychometric properties of the adult ADHD self report scale (ASRS) in hebrew. Isr J Psychiatry Relat Sci. (2010) 47:308–15. 21270505

[28] Kang KD Yun SW Chung U Kim TH Park JH Park IH. Effects of methylphenidate on body index and physical fitness in Korean children with attention deficit hyperactivity disorder. Hum Psychopharmacol Clin Exp. (2016) 31:76–82. 10.1002/hup.2514 26756111

[29] Mirkov D Nedeljkovic A Kukolj M Ugarkovic D Jaric S. Evaluation of the reliability of soccer-specific field tests. J Strength Cond Res. (2008) 22:1046–50. 10.1519/JSC.0b013e31816eb4af 18545209

[30] Masuda S Suganuma K Kaneko C Hoshina K Suzuki T Serita T. Prediction of falls using a 3-m Zigzag walk test. J Phys Ther Sci. (2013) 25:1051–4. 10.1589/jpts.25.1051 24259913 PMC3818753

[31] Aquino R Palucci Vieira LH De Paula Oliveira L Cruz Gonçalves LG Pereira Santiago PR. Relationship between field tests and match running performance in high-level young Brazilian soccer players. J Sports Med Phys Fitness. (2018) 58:256–62. 10.23736/S0022-4707.17.06651-8 28198600

[32] Neag I Mihaila I Fleancu LJ Stancu M Potop V Barbu D. Agility development in youth soccer: the efficacy of fixed-role small-sided games. Front Sports Act Living. (2025) 7:1593906. 10.3389/fspor.2025.1593906 40352681 PMC12061991

[33] Borg GAV Noble BJ. Borg’s perceived exertion and pain scales. Exerc Sport Sci Rev. (1974) 2:131–53. 10.1249/00003677-197400020-00006 4466663

[34] Banich MT Munakata Y. Modes of executive function and their coordination: introduction to the special section. Neuropsychologia. (2014) 62:319–20. 10.1016/J.NEUROPSYCHOLOGIA.2014.08.009 25132575 PMC12007443

[35] Friedman NP Robbins TW. The role of prefrontal cortex in cognitive control and executive function. Neuropsychopharmacology. (2022) 47:72–89. 10.1038/s41386-021-01132-0 34408280 PMC8617292

[36] Arnsten AFT. The emerging neurobiology of attention deficit hyperactivity disorder: the key role of the prefrontal association Cortex. J Pediatr. (2009) 154:I–S43. 10.1016/j.jpeds.2009.01.018 20596295 PMC2894421

[37] Cools R D’Esposito M. Inverted-U-shaped dopamine actions on human working memory and cognitive control. Biol Psychiatry. (2011) 69:e113–25. 10.1016/j.biopsych.2011.03.028 21531388 PMC3111448

[38] Alotaibi MM Stavrinos D Motl RW Bell M Snyder SW Hurt CP. Effect of psychostimulant medications on functional balance performance in persons with attention-deficit/hyperactivity disorder: a systematic review. Gait Posture. (2023) 102:146–58. 10.1016/J.GAITPOST.2023.03.019 37018889

[39] Bart O Daniel L Dan O Bar-Haim Y. Influence of methylphenidate on motor performance and attention in children with developmental coordination disorder and attention deficit hyperactive disorder. Res Dev Disabil. (2013) 34:1922–7. 10.1016/j.ridd.2013.03.015 23584172

[40] Soleimani R Kousha M Zarrabi H Mahnaz Tavafzadeh-haghi S Mohammad Jalali M. The impact of methylphenidate on motor performance in children with both attention deficit hyperactivity disorder and developmental coordination disorder: a randomized double- blind crossover clinical trial. Iran J Med Sci. (2017) 4242:354–61. PMC5523042 28761201

[41] Wang J Wang Y Ren Y. A case-control study on balance function of attention deficit hyperactivity disorder (ADHD) children. Beijing da xue xue bao Yi xue ban. (2003) 35:280–3. 12914246

[42] Hove MJ Zeffiro TA Biederman J Li Z Schmahmann J Valera EM. Postural sway and regional cerebellar volume in adults with attention-deficit/hyperactivity disorder. Neuroimage Clin. (2015) 8:422–8. 10.1016/J.NICL.2015.05.005 26106567 PMC4474325

[43] Alotaibi MM Motl RW Stavrinos D Snyder SW Singh H Lein DH. Effect of psychostimulant medications on static balance performance in adults with attention deficit hyperactivity disorder: within-subjects repeated-measure study. Hum Mov Sci. (2023) 88:103067. 10.1016/j.humov.2023.103067 36780727

[44] Plummer-D’Amato P Brancato B Dantowitz M Birken S Bonke C Furey E. Effects of gait and cognitive task difficulty on cognitive-motor interference in aging. J Aging Res. (2012) 2012:583894–8. 10.1155/2012/583894 23209905 PMC3503314

[45] Möhring W Klupp S Grob A. Effects of dual tasking and methylphenidate on gait in children with attention deficit hyperactivity disorder. Hum Mov Sci. (2018) 62:48–57. 10.1016/j.humov.2018.09.007 30243117

[46] Prieto-González P Sedlacek J. Effects of running-specific strength training, endurance training, and concurrent training on recreational endurance Athletes’ performance and selected anthropometric parameters. Int J Environ Res Public Health. (2022) 19:10773. 10.3390/IJERPH191710773 36078489 PMC9518107

[47] Tsuk S Zach S Glixman O Rotstein A Avieli E Mimouni-Bloch A. Attitudes toward stimulant medication for treating ADHD among physical education student teachers. J Phys Educ Sport. (2023) 23:1325–31. 10.7752/jpes.2023.05162

